# Eosinophil accumulation in postnatal lung is specific to the primary septation phase of development

**DOI:** 10.1038/s41598-020-61420-5

**Published:** 2020-03-10

**Authors:** Lucas F. Loffredo, Mackenzie E. Coden, Brian M. Jeong, Matthew T. Walker, Kishore Reddy Anekalla, Ton C. Doan, Raul Rodriguez, Mandy Browning, Kiwon Nam, James J. Lee, Hiam Abdala-Valencia, Sergejs Berdnikovs

**Affiliations:** 1 Division of Allergy and Immunology, Department of Medicine, Northwestern University Feinberg School of Medicine, Chicago, Illinois USA; 2 Division of Pulmonary and Critical Care, Department of Medicine, Northwestern University Feinberg School of Medicine, Chicago, Illinois USA; 3 Department of Biochemistry and Molecular Biology, Mayo Clinic Arizona, Scottsdale, USA

**Keywords:** Mouse, Eosinophils

## Abstract

Type 2 immune cells and eosinophils are transiently present in the lung tissue not only in pathology (allergic disease, parasite expulsion) but also during normal postnatal development. However, the lung developmental processes underlying airway recruitment of eosinophils after birth remain unexplored. We determined that in mice, mature eosinophils are transiently recruited to the lung during postnatal days 3–14, which specifically corresponds to the primary septation/alveolarization phase of lung development. Developmental eosinophils peaked during P10-14 and exhibited Siglec-F^med/high^CD11c^−/low^ phenotypes, similar to allergic asthma models. By interrogating the lung transcriptome and proteome during peak eosinophil recruitment in postnatal development, we identified markers that functionally capture the establishment of the mesenchymal-epithelial interface (*Nes*, *Smo*, *Wnt5a*, *Nog*) and the deposition of the provisional extracellular matrix (ECM) (*Tnc*, *Postn*, *Spon2*, *Thbs2*) as a key lung morphogenetic event associating with eosinophils. Tenascin-C (TNC) was identified as one of the key ECM markers in the lung epithelial-mesenchymal interface both at the RNA and protein levels, consistently associating with eosinophils in development and disease in mice and humans. As determined by RNA-seq analysis, naïve murine eosinophils cultured with ECM enriched in TNC significantly induced expression of Siglec-F, CD11c, eosinophil peroxidase, and other markers typical for activated eosinophils in development and allergic inflammatory responses. TNC knockout mice had an altered eosinophil recruitment profile in development. Collectively, our results indicate that lung morphogenetic processes associated with heightened Type 2 immunity are not merely a tissue “background” but specifically guide immune cells both in development and pathology.

## Introduction

In the adult healthy lung, there is only a small population of resident homeostatic eosinophils, totaling 1–2% of all CD45^+^ hematopoietic cells^1,2^. Increased eosinophil numbers and shifts in eosinophil phenotype and function are normally associated only with active recruitment to the inflamed lung during infection or allergic disease, such as asthma^1,3^. In disease, eosinophils are traditionally perceived to have detrimental effector functions, causing lung tissue damage via release of their cytotoxic granule cationic proteins stimulated by allergens, helminth infection, or tissue injury^4^. However, it has been increasingly appreciated that subsets of these cells have various homeostatic functions in non-inflammatory contexts. In health, eosinophils are associated with stem cell activity and normal morphogenetic and repair processes at various tissue sites, summarized by James Lee *et al*. in his LIAR (*L*ocal *I*mmunity *A*nd/or *R*emodeling/Repair) hypothesis^5^. Eosinophil resident populations can be found in different organs, such as the thymus, gastrointestinal tract, and uterus^6–8^. One common feature of these tissue sites is a high degree of epithelial turnover and activity of morphogenetic programs during postnatal development. Some of the developmental and morphogenetic events that strongly associate with high eosinophil activity are the branching duct morphogenesis of mammary glands, epithelial shedding and turnover in the small intestine, and smooth muscle proliferation^5,9–11^.

Despite the widespread notion that the mammary gland is the only body organ to undergo significant branching morphogenesis postnatally^12^, the lung similarly continues its branching morphogenesis and development after birth^13^. The end of the saccular morphogenetic stage, as well as primary and secondary septation during lung alveolarization, all take place in the postnatal phase of lung development^14^. The lungs of newborn mice are characterized by increased expression of epithelial-derived cytokine IL-33 and increased numbers of group 2 innate lymphoid cells (ILC2s), previously thought to be induced by the first environmental stress to the airway at birth^15^. ILC2s are thought to drive Type 2 immunity in the neonatal lung, which associates with Type 2 cytokine production (IL-5 and IL-13), M2 macrophage polarization, and temporal increases in eosinophil numbers^15–17^. However, how these transient events relate to normal lung developmental programs is currently unknown. Despite the established link of eosinophils and other innate cells to morphogenetic events, it remains entirely unexplored whether the neonatal presence of eosinophils in the lung is potentially driven by normal lung morphogenesis.

The reciprocal relationship between developing tissues and cells of the innate immune system is poorly understood, and tissue-specific processes and factors attracting and engaging eosinophils in both normal development and disease are currently unknown. Although there is two-way communication between tissues and immune cells, in this study we aimed to characterize lung tissue developmental processes underlying heightened Type 2 responses rather than the role that eosinophils play in lung development. Specifically, we: (1) carefully dissected the recruitment kinetics of eosinophils relative to key developmental stages of the neonatal murine lung; (2) determined that peak eosinophil presence in the developing lung (postnatal days 10–14) corresponds to the primary septation of the lung during the alveolarization phase of development, characterized by the formation of mature epithelium; (3) identified key morphogenetic signals, in particular the deposition of tenascin-C (TNC), that specifically align with timing of lung eosinophil recruitment; (4) found that extracellular matrix enriched with TNC is sufficient to induce eosinophil activation consistent with the phenotype induced by development or lung allergic inflammation. In summary, eosinophil recruitment to the lung during postnatal development (modeling) and allergic disease (re-modeling) may represent an inherently homeostatic response of the innate immune system driven by morphogenesis of the airway epithelium.

## Materials and Methods

### Mice

Wild-type C57BL6 mice were purchased from Jackson Laboratories. Tenascin-C knockout (TNC−/−) mice (C57BL/6 N-TgH) were from RIKEN, Japan. PHIL mice originated from Lee Laboratories (Mayo Clinic Arizona). Experiments were conducted at different time points postnatally, with “P0” indicating postnatal day 0, “P3” indicating postnatal day 3, *et cetera* (including tissue harvest days P7, P10, P14, P18, P22, P26, P30, and P34). At least 2 separate litters of mice were utilized for lung flow cytometry or gene expression at each of the time points. 6–8 week old wild-type C57BL6 mice were utilized for bone marrow-derived eosinophil cultures. All animal experiments were approved by Northwestern University’s Institutional Animal Care and Use Committee (IACUC). All methods involving mice were performed in accordance with relevant guidelines and regulations.

### Bone marrow-derived eosinophil culture

Bone marrow-derived eosinophils (BMEos) were cultured as previously described^18^. Briefly, bone marrow was extracted from the femurs and tibias of 6–8 week old wild-type mice and plated at 1 million cells/ml in media composed of RPMI-1640, HEPES buffer, nonessential amino acids, and sodium pyruvate (Corning); glutamine and penicillin/streptomycin (HyClone); 2-mercaptoethanol and 20% heat-inactivated fetal bovine serum (Sigma). From days 0–4 of culture, FLT3-L and SCF (Peprotech) at 100 ng/ml each were added to the media to expand the precursor pool. At day 4 and every other day afterwards, the media was replaced and cells were re-plated at 1 million cells/ml, with 10 ng/ml of IL-5 (Peprotech) added to the media each time. By day 13, cultures consisted of >90% live and pure eosinophils as determined by flow cytometry.

### Flow cytometry and eosinophil sorting from lung tissue

Prior to harvest and homogenization, bronchoalveolar lavage fluid was collected and the lungs were perfused with ice cold PBS through the right heart ventricle. The left side of the lung and the right inferior and mediastinal lobes were used for flow cytometry. Lungs were dissociated in 0.2 mg/ml DNAse I (Roche) and 2 mg/ml Collagenase D (Roche) for 1 hour. The cells were then filtered into a single cell suspension using a sterile mesh. Red blood cells were lysed with PharmLyse RBC lysis buffer (BD). 5 million cells were utilized for flow cytometry staining. Prior to antibody staining, cells were incubated with Zombie Live/Dead Aqua (Biolegend) dye followed by CD16/32 FC Block (BD Pharmingen). We used the following antibody cocktail to assess leukocyte populations in lung development: (1) FITC-conjugated CD45 (clone 30-F11, Biolegend); (2) APC-Cy7-conjugated CD11b (clone M1/70, BD); (3) PE/Cy7-conjugated CD11c (clone N418, Biolegend); (4) Alexa Fluor 647-conjugated Siglec-F (clone E50-2440, BD); (5) PE-conjugated CD64 (clone X54-5/7.1.1, BD); (6) Alexa Fluor 594-conjugated CD3 (clone 17A2, Biolegend); (7) PerCP-Cy5.5-conjugated CD19 (clone eBio1D3, eBioscience); (8) eFluor450-conjugated Ly-6C (clone HK1.4, eBioscience); and (9) Alexa Fluor 700-conjugated Ly-6G (clone 1A8, Biolegend). Cells were then fixed in 2% paraformaldehyde and analyzed on an LSRII flow cytometer (BD). Compensation was set up using single color control fluorescent beads (OneComp, eBioscience; and ArC, Molecular Probes). Negative gate boundaries were identified using fluorescence-minus-one (FMO) controls. FlowJo software (Treestar) was used for compensation and data analysis. Eosinophils were gated as CD45^+^CD11b^+^CD64^−^Ly6G^+/−^Ly6C^−^CD11c^−/low^Siglec-F^med/high^. BALs extracted from mice at P10 or older were analyzed by flow cytometry using the same cocktail and staining protocol. Bone marrow-derived eosinophils were stained with the following antibody combination: (1) Alexa Fluor 700-conjugated CD45 (clone 30-F11, Biolegend); (2) APC-Cy7-conjugated Siglec-F (clone E50-2440, BD); (3) PE/Cy7-conjugated Ly-6A/E (Sca-1) (clone D7, Biolegend) and (4) PE Dazzle 594-conjugated CD117 (c-kit) (clone 2B8, Biolegend). The RNA-Seq data generated utilized eosinophils sorted from the lungs of naïve and ovalbumin-challenged mice. See the publication by Abdala-Valencia *et al*.^1^ for a detailed description of the ovalbumin model of asthma and eosinophil sorting protocol.

### Assessment of eosinophil morphology

To further confirm detection of eosinophils in postnatal lungs by flow cytometry, 20,000 cells from the BALs of mice at postnatal day 10 were mounted on slides using cytospins, dried, and fixed in Wright Giemsa Solution (EMD Millipore) for 2 minutes. Differential Quik Stain Kit (Electron Microscopy Sciences) was used to differentially stain eosinophils. Eosinophils were identified by their characteristic ring-shaped nuclei and red eosin staining of granules. Microscopy images were taken using an Olympus IX71 confocal microscope and cellSens Imaging Software (Olympus).

### Quantitative PCR (qPCR)

The lung tissue for gene expression analysis was harvested from the developing lungs of the same mice that were assessed for eosinophil recruitment by flow cytometry. Tissue was stored in RNALater (Qiagen) solution prior to RNA extraction. Approximately 20 mg of tissue was homogenized using a rotor homogenizer and RNA was extracted with the RNeasy Mini Kit (Qiagen). 500 ng of total RNA was used in a cDNA synthesis reaction, using a cDNA Synthesis Kit (Quanta). qPCR reactions utilized probe-based qPCR Master Mix (IDT), and pre-designed primer/probe gene expression assays (IDT) for the following genes (targeted exons indicated in parentheses): Nes (exon 2–3), Smo (exon 8–9), Wnt5a (exon 5–6), Nog (exon 1–1), Tnc (exon 10–11), Spon2 (exon 4–5), Postn (exon 2–3), Thbs2 (exon 13–14), and Adam33 (exon 3–5). qPCR reactions were run using a StepOnePlus Real-Time PCR System (Applied Biosystems). Gene expression was calculated relative to expression of GAPDH (housekeeping gene) and was reported as true copies of the gene of interest per 10^4^ copies of GAPDH as previously described^19^.

### Secondary analysis of publicly available microarray and proteomics data

NCBI’s GEO Datasets and LungMAP repositories were used to mine publicly available gene and protein signatures of interest. For re-analysis of raw data, we used the Series Matrix Files containing normalized expression values for the microarray datasets used in this study. The R Bioconductor package was used to assess differential expression between groups of interest in these datasets. For murine miRNA and mRNA profiling of 7 time points, GSE20954 was used. For murine gene expression profiles using the Swiss-Webster Strain Dataset, the LungGens LungDTC repository was used. The LungGens LungDTC repository was used to mine MicroArray data from dataset GSE74243 (murine gene expression data for 10 pre- and post-natal time points in three laboratory mice strains). The LungMAP database was used to mine murine lung proteomics data that was generated at the University of Alabama at Birmingham (PI: Namasivayam Ambalavanan). For RNA-seq gene expression data from human lung development, the LungGENS LungSortedCells repository was used. LungGENS data was generated by the LungMAP Human Tissue Core: Biorepository for Investigation of Neonatal Diseases of Lung-Normal at the University of Rochester Medical Center (PI: Gloria S. Pryhuber). The LungGENS samples were analyzed from 12 LungMAP donor subjects from 1 day old to adult. Immune cells were sorted as CD45^+^, epithelial cells were CD45^−^PECAM^+^VE-Cadherin^−^EpCAM^+^, endothelial cells were CD45^−^PECAM^+^VE-Cadherin^+^, and mesenchymal cells were CD45^−^PECAM^−^VE-Cadherin^−^EpCAM^−^.

### RNA-Seq

Terminal BMEos (cultivated as described above) were plated at 500,000 cells/ml with either media alone, media with 500 μg/ml ECM gel (Sigma-Aldrich), or media with ECM gel combined with 25 μg/ml of Tenascin-C peptide representing the fibronectin type III binding domain (Kollodis Biosciences; motif: VAEIDGIEL). Cells were incubated for 24 hours, then all cells were lysed and RNA was immediately extracted using the RNeasy Plus Mini Extraction Kit (Qiagen). RNA quality was assessed using an Agilent High Sensitivity RNA ScreenTape System (Agilent Technologies). The NEBNext Ultra RNA Library Prep Kit for Illumina (New England Biolabs) was utilized for full-length cDNA synthesis and library preparation. The sequencing of cDNA libraries was done on an Illumina NextSeq. 500 instrument (Illumina) at a target read depth of approximately 10 million aligned reads per sample. The pool was denatured and diluted to create a 2.5 pM DNA solution. The PhiX control was spiked at 1%. The pool was then sequenced by 1 × 5 cycles using the NextSeq. 500 High Output Kit (Illumina). Sequenced reads were demultiplexed using bcl2fastq (v 2.17.1.14). Quality control was performed using FastQC. Low quality reads were discarded using trimmomatic (v 0.33). Reads were aligned using TopHat2 to mouse reference genome mm10. Read counts were generated using htseq-count. Differential expression analysis was done using edgeR (R/Bioconductor package). All the computational analysis was performed on genomic nodes of Quest, Northwestern’s High Performance Computing Cluster.

### Bioinformatics, statistical analysis, and data visualization

Gene set enrichment analysis was performed using GeneGO Metacore software (Thomson Reuters) to access GO Biological Processes represented in gene expression signatures, produced either from data mining or our RNA-Seq analysis. Gene-biological process functional integration networks were constructed using the Cytoscape 3.2 software with the ClueGO functional analysis plug-in (v 2.2.6)^20^. Venn diagram analysis was used to identify the intersections of gene signatures from multiple studies, using the Bioinformatics & Evolutionary Genomics Venn diagram tool (VIB/UGent, Belgium). Heatmap generation and hierarchical clustering were done using the “gplots” package in R. Statistical tests utilized in different experiments are identified in the captions of corresponding figures and tables. All graphs and statistical analyses were carried out in GraphPad Prism (Graphpad) and Systat 13 (Systat Software, Inc.). Flow cytometry graphs were generated using FlowJo (Treestar). Principal components analysis was performed using the multivariate statistical analysis software PAST, Version 3.0.

## Results

### Eosinophils peak between postnatal days 10 and 14 during the primary septation stage of normal murine lung development

**Figure 1:**
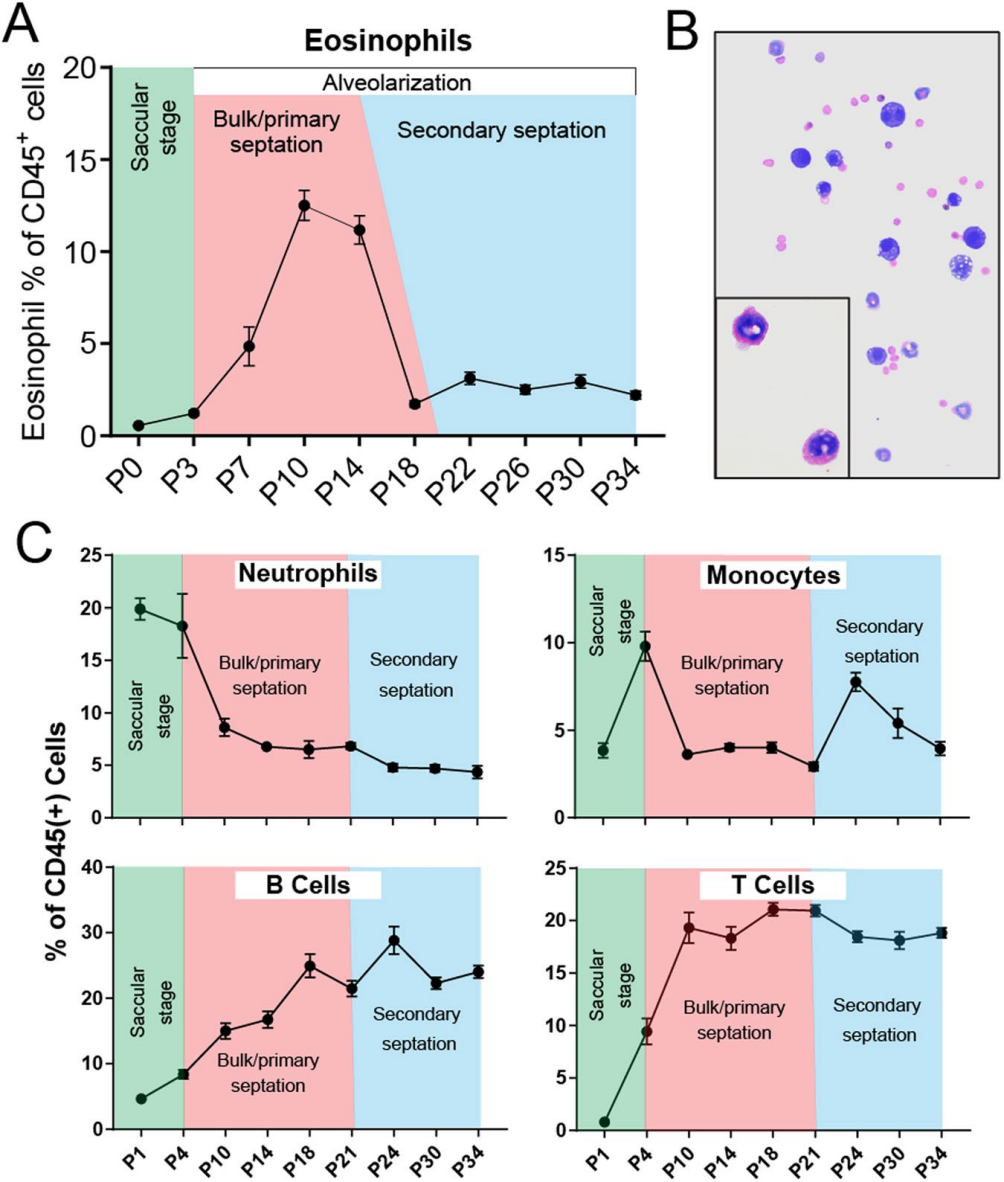
Transient recruitment of eosinophils to the murine lung during normal postnatal development. (**A**) Kinetics of lung eosinophil recruitment during normal postnatal development. The peak of eosinophil recruitment coincides with bulk/primary septation during the alveolarization phase of lung development. N = 4–8 mice/group, combined by different litters. Eosinophils are quantified as a percentage of all CD45^+^ hematopoietic cells. (**B)** Cytospin preparation of bronchoalveolar lavage from normal murine lungs during day 10 of postnatal development. Zoom-in box shows developmental eosinophil biology. (**C)** Kinetics of lung immune cell recruitment during normal postnatal development. The peak of non-eosinophil immune cell recruitment does not coincide with eosinophil recruitment in the bulk/primary septation phase. N = 4–8 mice/group, combined by different litters.

To carefully dissect the kinetic of eosinophil recruitment during postnatal lung development, we used multi-color flow cytometry to analyze healthy mouse lung tissue from birth until P34 days postnatally (adulthood) at 3–4 day intervals (Fig. 1). By P7, the numbers of eosinophils in the developing lung began to rise, reaching peak numbers during the P10-P14 developmental window. At these time points, eosinophils constituted 10–15% of all CD45^+^ hematopoietic cells, which is equivalent to eosinophil recruitment to the lung in a 3-challenge ovalbumin model of asthma (Fig. 1A). The same pattern is seen by total eosinophil counts (Fig. S1). Differential staining of cells isolated from the bronchoalveolar lavage of P10 developing lungs confirmed an increased presence of mature eosinophils positive for eosin staining that exhibit nuclear “donut-shape” and vacuolarized (vEos) morphology (Fig. 1B). After P14, eosinophil levels rapidly declined, returning to the sustained low homeostatic levels typical for adult tissue by P18 (Fig. 1A). To confirm that this kinetic is specific to eosinophils, we looked at the recruitment of other immune system cell types in the same time frame of postnatal development. While neutrophils, monocytes, B cells, and T cells showed kinetic responses throughout lung development, none matched the specific peak of eosinophil recruitment during the P10–14 window (Fig. 1C). We next assessed whether this eosinophil kinetic corresponds to particular stages of lung development. The lung is one of the few organs that continues morphogenesis after birth, both in humans and mice. Postnatal murine lung development lasts until day P28 and is characterized by three distinct developmental stages^13^. P0–P3 marks the end of the saccular stage, succeeded by lung alveolarization occurring between P3 and P14 (bulk/primary septation) and P14 into adulthood (secondary septation)^13^. Eosinophil presence in the developing lung specifically matches the primary septation stage during alveolarization (Fig. 1A), which is marked by epithelial barrier formation along newly created alveoli. In summary, eosinophil recruitment in postnatal lung development occurs between P3 and P18, peaking at P10-14, and specifically corresponds to the bulk/primary septation stage of the lung alveolarization phase.

### Kinetics of lung tissue marker expression confirm peak mesenchymal activity and provisional matrix deposition as key developmental events during eosinophil recruitment window

**Figure 2:**
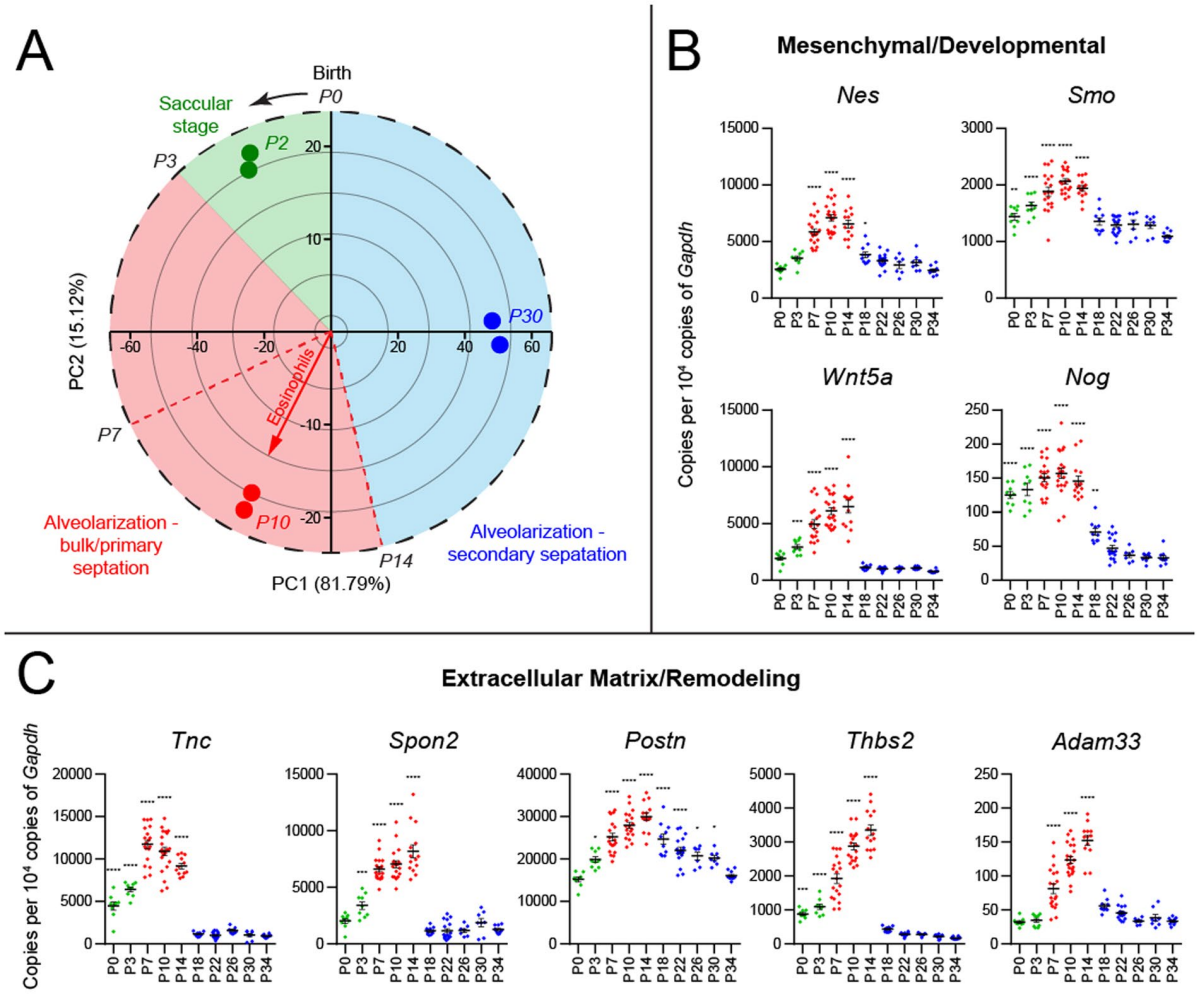
Lung tissue gene expression and proteome profiles corresponding to the kinetics of eosinophil recruitment during normal lung postnatal development. (**A**) Principal component analysis of changes in the lung tissue transcriptome over the course of postnatal lung development (postnatal days 0 to 34). On this “developmental clock”, eosinophils peak at postnatal day 10, which represents the alveolarization and bulk/primary septation stage of lung development. (**B)** Expression of mesenchymal and developmental pathway genes during postnatal development. (**C)** Expression of extracellular matrix and tissue remodeling genes during postnatal development. *Green*: saccular stage of lung development. *Red*: alveolarization (bulk/primary septation). *Blue*: alveolarization (secondary septation). ****p < 0.0001, one-way ANOVA with Dunnett’s multiple comparisons test.

In order to further pinpoint specific lung tissue markers corresponding to peak eosinophil recruitment in development, we performed a secondary analysis of the transcriptomics data set (NCBI GEO, GSE20954) addressing lung gene expression throughout postnatal development. An unbiased PCA approach let us reduce the dimensionality of data and summarize the developing lung transcriptome, further mapping it to developmental stages during the P0 to P30 time window (Fig. 2A). We then used this “developmental clock” to mine genes with the highest loadings in the direction of variation corresponding to the P7–P14 eosinophil recruitment window (Fig. 2A, arrow). We further validated developmental stage-dependent expression of these markers using qPCR analysis of murine lungs at 3–4 day intervals throughout postnatal development (P0 to P34), carefully timing birth events and using mice from different litters to represent each developmental time point (Fig. 2B,C). Expression of mesenchymal and provisional extracellular matrix proteins matched exactly the eosinophil recruitment profile. Some representative genes included markers of mesenchymal stem cells (nestin (*Nes*)) and developmental pathways known to regulate epithelial differentiation (Smoothened (*Smo*), WNT5a (*Wnt5a*), and Noggin (*Nog*)) (Fig. 2B). Deposition of the provisional extracellular matrix during P7-P14 was strongly represented by peak expression of tenascin-C (*Tnc*), spondin 2 (*Spon2*), periostin (*Postn*), thrombospondin 2 (*Thbs2*), and the marker of remodeling, *Adam33* (Fig. 2C).

### Only gene expression of mesenchymal and provisional matrix proteins peaks during the alveolarization phase of development

**Figure 3:**
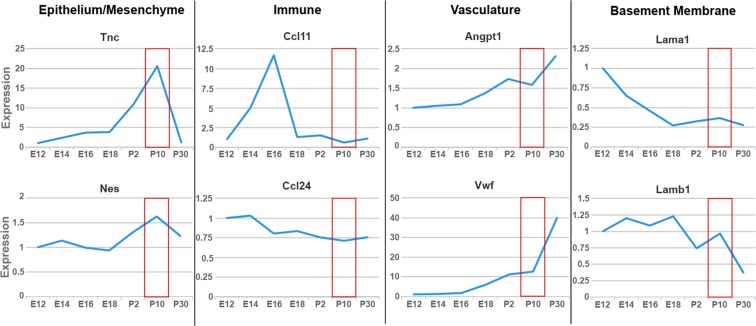
Peak recruitment of eosinophils to the lung specifically aligns with peak expression of provisional matrix and mesenchymal genes rather than genes representing immune and vascular compartments. Only mesenchymal and provisional matrix genes (exemplified here by *Tnc* and *Nes*) peak in expression during alveolarization, the phase denoted by the red box. Other proteins related to eosinophils or lung development do not peak during this time (LungGens data mining).

To confirm our findings from Fig. 2B that tissue genes corresponding to eosinophil recruitment specifically represent the epithelial/mesenchymal compartment, we mined lung development gene expression data from the LungGens LungDTC repository. Using an independent data set, we further validated that expression of mesenchymal and provisional extracellular matrix proteins *Tnc* and *Nes* peaked during primary septation in postnatal development. However, other genes, such as eotaxins *Ccl11* and *Ccl24* (immune compartment), endothelial-specific genes *Angpt1* and *Vwf* (vascular compartment), and laminins *Lama1* and *Lamb1 (*basement membrane constituents*)*, did not have peak expression during this specific developmental phase (Fig. 3).

### Provisional matrix protein tenascin-C is deposited in the lung both during development and allergic inflammation

**Figure 4:**
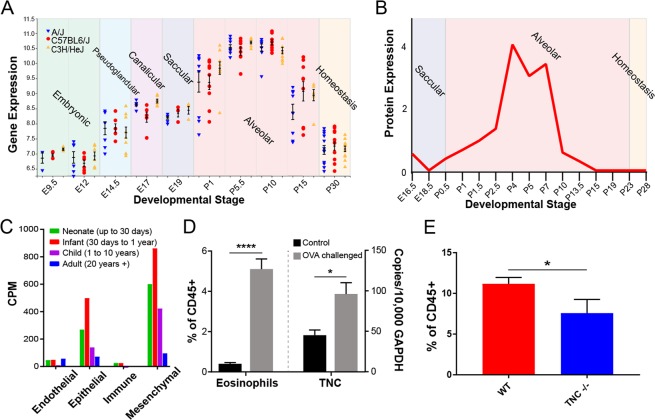
Tenascin-C is robustly expressed in lung development and mouse models of asthma. (**A**) Gene expression of TNC in three different strains of mice over the course of murine prenatal and postnatal development (LungMAP data mining). (**B)** Protein levels of TNC during murine postnatal development (LungMAP data mining). (**C)** Human lung expression of TNC in epithelial and mesenchymal cells (LungMAP human lung next generation sequencing). (**D)** Detection of eosinophils by flow cytometry and lung tissue expression of TNC in a mouse model of asthma. *p < 0.05, ****p < 0.0001, unpaired T-test. (**E)** Eosinophil recruitment during normal postnatal development in wild-type mice (red) compared to TNC−/− mice (blue). N = 4–8 mice/group, combined by different litters. Eosinophils are quantified as a percentage of all CD45^+^ hematopoietic cells. *p < 0.05, unpaired T-test.

Our study identified this ECM provisional glycoprotein as the key marker paralleling tissue eosinophil expansion during the primary septation phase of lung development. Throughout the course of murine lung development, the expression of TNC peaks after birth specifically during the alveolarization phase (Fig. 4A). This TNC kinetic was very consistent in three different strains of mice (C57BL6/J, A/J and C3H/HeJ) (Fig. 4A) and exhibited a similar pattern at the protein level (Fig. 4B). TNC is one of the markers of human asthma^21,22^. In human lungs, we found that TNC expression was restricted to epithelial and mesenchymal cells, and its expression was highest in neonates (up to 30 days after birth) and infants (30 days to 1 year) (Fig. 4C). This time frame in human lung development specifically corresponds to the alveolarization phase^23,24^, which perfectly matches the mouse time course of TNC expression in development. We further confirmed this in an independent model of asthma by demonstrating the expression of TNC in lung tissue of allergic mice that had significantly increased recruitment of eosinophils (Fig. 4D). Furthermore, we looked at the recruitment of eosinophils to the lung during the alveolarization phase at postnatal day 14 in wild-type and TNC knockout mice. We found that in the absence of TNC, eosinophil recruitment is significantly reduced compared to wild-type mouse lung development (Fig. 4E), which suggests a regulatory role for TNC in eosinophil biology both during development and inflammation. Other cell types, including B and T Cells had significantly increased recruitment in this model as well, and monocytes displayed significantly reduced recruitment (Supplementary Fig. 2), likely as a consequence of altered tissue microenvironment in absence of TNC.

### Extracellular matrix induces profound changes in the gene expression profiles of naïve eosinophils

**Figure 5:**
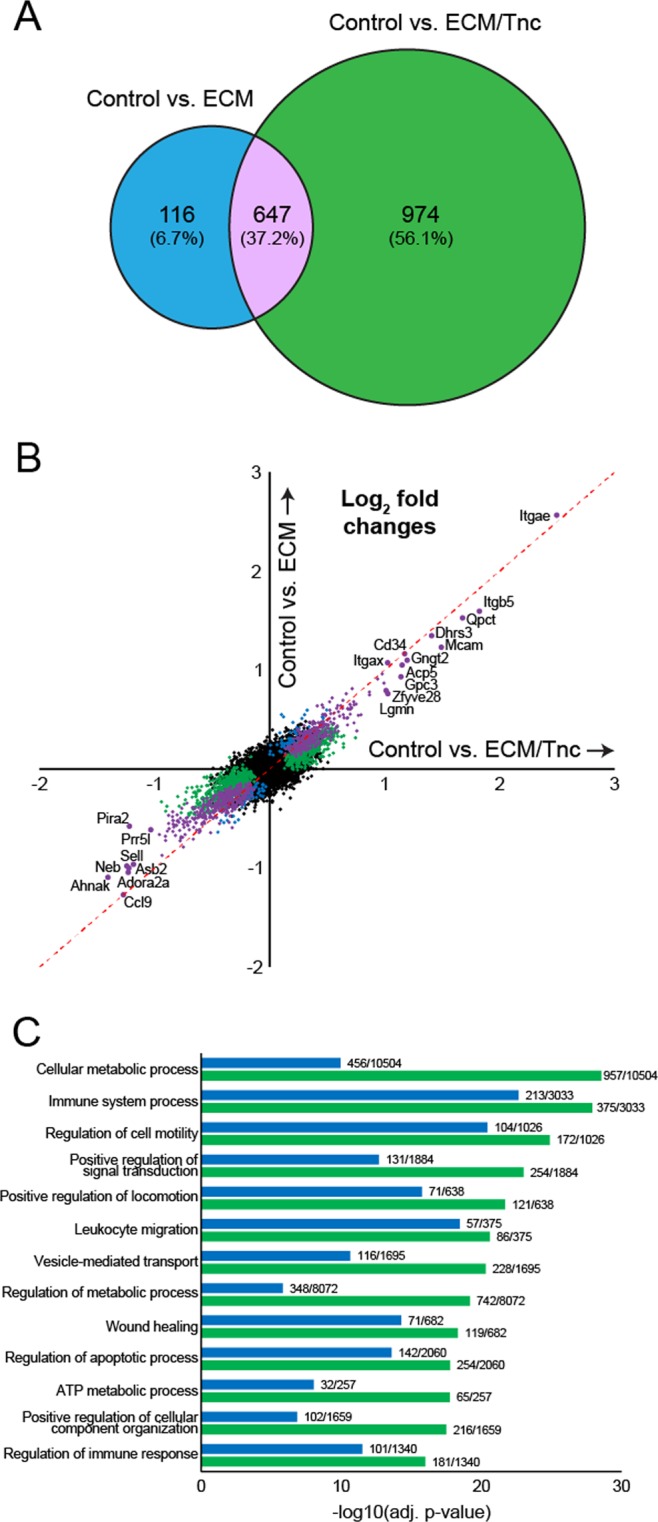
RNA-seq analysis of gene expression profiles commonly represented in eosinophils interacting with homeostatic vs. provisional extracellular matrix environments. (**A**) Venn diagram showing numbers of differentially expressed genes in naïve eosinophils stimulated with matrigels vs. matrigels enriched with tenascin C. Tenascin C treated matrigels triggered a significantly larger response from naïve eosinophils. (**B)** Correlation between gene expression profiles induced by ECM vs. ECM/TNC treatment. *Blue*: genes uniquely upregulated by “homeostatic” ECM. *Green*: genes uniquely overexpressed by TNC. *Purple*: the 647 genes commonly expressed by both treatments (overlap shown in part A). (**C)** Biological processes represented by differential gene expression signatures identified in RNA-Seq analysis. Similar biological responses were triggered in eosinophils by both treatments, although overrepresented by a higher number of genes in the ECM/TNC treatment group. Regulation of metabolism, vesicle transport, and cellular structure organization were mostly overrepresented in ECM/TNC gene signatures. N = 4/group, 2 experimental repeats, p_adj_ < 0.05, Benjamini-Hochberg moderated t-test.

Given such robust expression and role for TNC in the regulation of eosinophil recruitment to lung tissue, we performed an RNA-Seq analysis of naïve murine bone marrow-derived eosinophils cultured in the presence of the homeostatic or provisional (TNC-enriched) matrix for 24 hours (Fig. 5) to simulate lung environments experienced by eosinophils in homeostasis and development/inflammation. 763 genes were significantly differentially expressed (adjusted p-value < 0.05) by the interaction of eosinophils with the homeostatic matrix, while 1621 genes were significantly changed by interaction with the provisional ECM/TNC matrix (Fig. 5A). 647 genes (37.2%) were commonly differentially expressed by interaction with either ECM microenvironment (Fig. 5A). Shared genes typically exhibited the highest fold changes values, although fold changes were the highest in TNC-enriched eosinophil environment (Fig. 5B). Likewise, GO biological processes represented by differentially expressed genes were similar in both ECM treatments, although represented by significantly higher number of genes in TNC-enriched treatment (Fig. 5C). Among these, regulation of metabolism and vesicle-mediated transport were represented disproportionately higher by gene signatures induced by the provisional ECM environment (Fig. 5C). The original gene expression data is provided in Supplementary Tables 1, 2. We further profiled TNC-specific gene signatures by directly comparing the gene expression of naïve eosinophils induced by “homeostatic” ECM vs. gene expression induced by TNC-enriched ECM, using RNA-Seq (Supplementary Fig. 3). 91 genes were differentially expressed between two ECM conditions at FDR-adjusted p-value < 0.01, 38 downregulated and 53 upregulated (Supplementary Fig. 3). A functional network analysis of this signature revealed that the exposure of eosinophils to ECM enriched with TNC results in biological processes associated with the regulation of leukocyte differentiation and apoptosis, as well as alterations in metabolism and mitochondrial behavior (Supplementary Fig. 3). Functional validation of these processes (summarized in Supplementary Fig. 3) in murine eosinophil cultures determined that in comparison with more homeostatic ECM exposures, interaction with a TNC-enriched provisional matrix promotes viability and controls the maturational state of eosinophils in hematopoietic conditions. Collectively, the provisional matrix promotes a higher transcriptional activity from naïve eosinophils, as well as features the activation of a distinct subset of genes with a more unique biological function.

### Extracellular matrix is sufficient to upregulate markers of tissue-activated eosinophils

**Figure 6:**
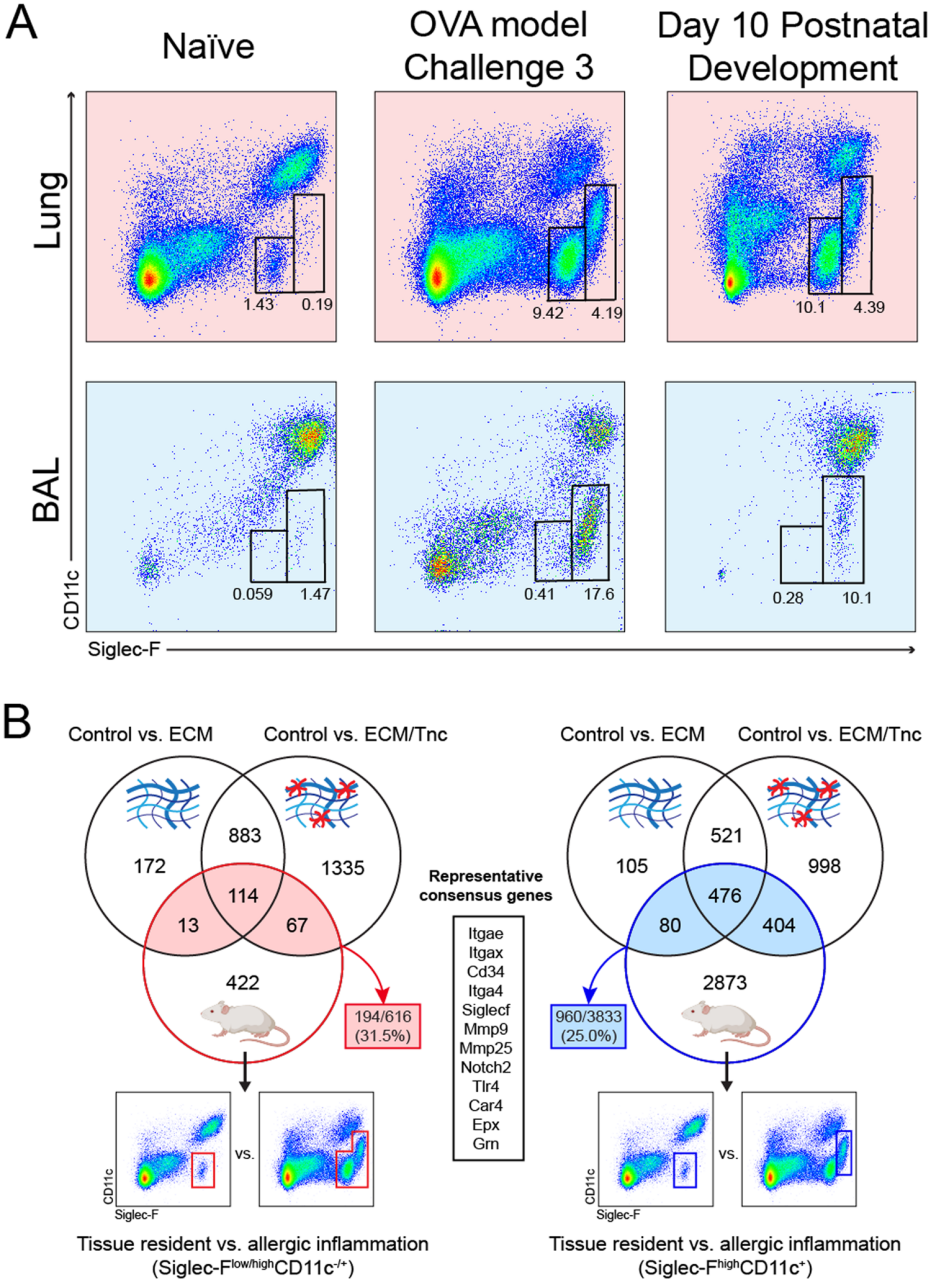
Comparison of gene signatures of bone marrow-derived eosinophils activated by interaction with the extracellular matrix *in vitro* against genes differentially expressed by lung eosinophils during allergic inflammation *in vivo*. (**A**) Flow cytometry showing eosinophil populations in representative lung tissue homogenates and bronchoalveolar lavage from normal adult mouse lungs, lungs of mice subjected to allergen challenge in an ovalbumin model of asthma, and normal mouse lungs harvested at day 10 during postnatal development (left to right). Representative samples are shown. (**B)** *Venn diagrams, black circles:* matrix-induced differentially expressed gene signatures from Fig. 6. *Venn diagrams, colored circles*: differential gene expression (relative to the gene expression of resident “naïve” lung tissue eosinophils) of total eosinophil population (left, red, secondary microarray analysis) or Siglec-F^high^CD11c^+^ eosinophils (right, blue, in-house RNA-Seq analysis of tissue-isolated eosinophils) sorted from murine lung tissue during allergic inflammation (3 challenge ovalbumin model of asthma). Mouse images within circles were created with BioRender.com under the academic license. Flow cytometry charts show the gating strategy for the sorting of corresponding eosinophil populations from lung tissue. Colored arrows and text boxes demonstrate the degree of overlap between ECM- and inflamed lung tissue-induced eosinophil gene activation signatures. Black text box in the middle lists several key representative genes shared by eosinophils in all comparisons.

Phenotypically, lung tissue eosinophils at P10 in development were similar to lung eosinophils in a model of allergic inflammation, represented by both Siglec-F^med^CD11c^−^ and Siglec-F^high^CD11c^+^ populations in the lung interstitium, as well as Siglec-F^high^CD11c^+^ population in the bronchoalveolar lavage fluid (Fig. 6A). Moreover, our RNA-seq results in Fig. 5 demonstrated that naïve eosinophils interacting with ECM upregulated markers typical of tissue-activated eosinophils in lung allergic inflammation, such as *Itgax* (CD11c), *Siglecf* (Siglec-F), *Car4* (carbonic anhydrase 4), *Itgae* (CD103), and *Cd34* (CD34). This prompted us to compare the gene signatures of ECM-stimulated eosinophils with the gene expression profiles of eosinophils sorted from lung tissues in a murine model of asthma (Fig. 6B). Remarkably, when we compared gene expression profiles of either ECM or ECM/TNC stimulated eosinophils (RNA-seq data in Fig. 5) with the gene expression profile of eosinophils sorted from lungs in a murine model of asthma (GSE57757, GEO, NCBI), we found that 194 out of 616 eosinophil genes in a model of asthma matched the response triggered by the ECM (Fig. 6B, left). Among these, 13 convergent genes were induced by “homeostatic” ECM and 67 genes by “provisional” ECM. We further refined this approach by sorting a specific population of Siglec-F^high^CD11c^+^eosinophils that are known to associate with trans-epithelial recruitment, fibrinogen adhesion, and a tissue-activated phenotype^1^. We contrasted the gene expression profile of this eosinophil population vs. the tissue-resident Siglec-F^low^CD11c^−^ population by RNA-Seq. 960 genes from this differentially expressed signature aligned with the gene expression signatures of eosinophils activated by the ECM (Fig. 6B, right). 404 genes specifically aligned with the eosinophil signature induced by an ECM/TNC interaction, while only 80 genes uniquely intersected with “homeostatic” ECM activation. 476 genes commonly intersected with either ECM treatment. Genes commonly expressed on eosinophils after ECM exposure *in vitro* or tissue activation during Th2 inflammation *in vivo* are consistent with eosinophil markers known to represent tissue-activated states (among them, *Itgax*, *Itgae*, *Siglecf*, *Mmp9*, *Tlr4*, *Car4*, and *Epx*) (Fig. 6B, middle box). In summary, the interaction of naïve murine eosinophils with the ECM, in the absence of additional cytokine or chemokine stimulation, is sufficient to activate transcriptional programs consistent with eosinophil tissue-activated states during allergic inflammation.

### Eosinophil deficiency results in altered extracellular matrix and mesenchymal gene expression in lung tissue

**Figure 7:**
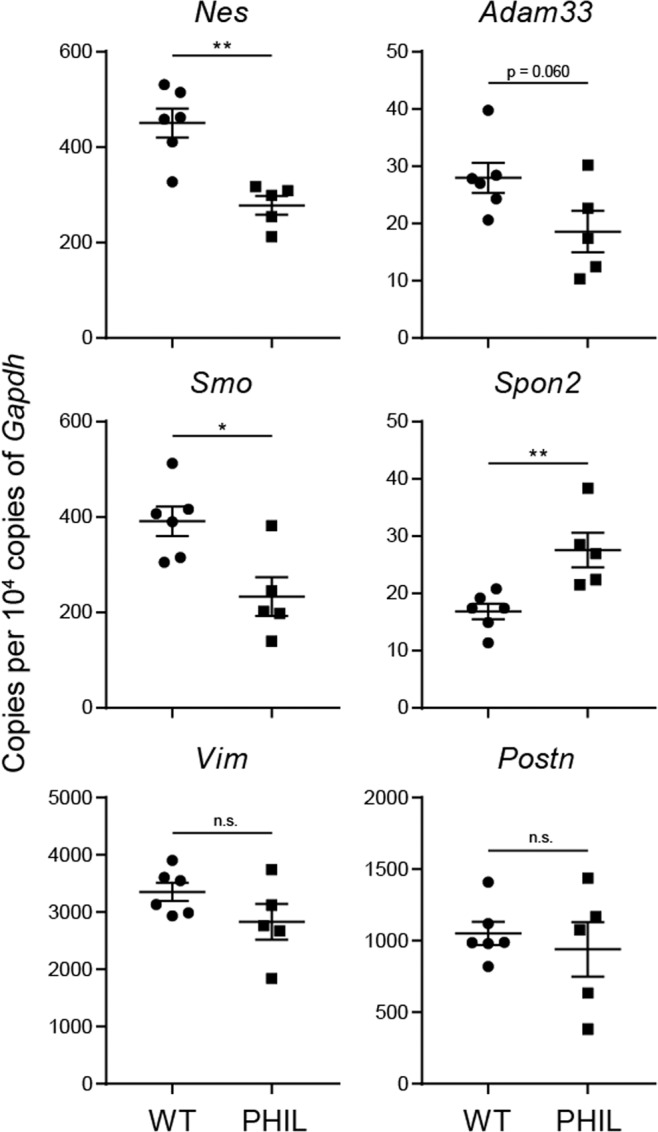
Lung tissue gene expression differences between eosinophil-deficient and control mice. *WT*: wild-type mice. *PHIL*: mouse strain with constitutive eosinophil deficiency. *p < 0.05; **p < 0.01; n.s., not significant, unpaired t-test.

Our results demonstrate that the non-immune lung tissue microenvironment in development and allergic remodeling is a critical factor in shaping eosinophil identity and function. To provide further insights into whether eosinophil-tissue interaction is a two-way functional relationship, we interrogated whether eosinophil depletion would have consequences for the developmental patterning of lung tissue. For this, we used PHIL mice, a strain constitutively lacking eosinophils, to compare mesenchymal and matrix gene expression between the lung tissue of eosinophil-deficient and wild-type mice by quantitative PCR (Fig. 7). In lung tissue at a steady state, we found a consistent significant downregulation of the mesenchymal marker nestin (*Nes*) in PHIL mice compared to wild-type controls, as well as the downregulation of developmental marker Smoothened (*Smo*) and a marginal decrease in vimentin (*Vim*), another mesenchymal marker (Fig. 7). Moreover, we found changes in the gene expression of mindin (spondin 2, *Spon2*), and disintegrin and metalloproteinase domain-containing protein 33 (*Adam33*) (Fig. 7). These markers also show an association with eosinophils in normal lung postnatal development (Fig. 2). Collectively, this suggests that eosinophils are not only directly instructed by the lung tissue compartment, but are also active participants in the maintenance of lung tissue homeostasis.

## Discussion

Our work demonstrates the critical significance of the immediate non-immune tissue microenvironment in regulating the phenotype, function, and accumulation of eosinophils. We show that: (1) eosinophil participation in normal postnatal lung morphogenesis is restricted to the bulk septation and alveolarization phases of development; (2) eosinophils specifically associate with the deposition of the provisional ECM and mesenchymal developmental activity; (3) the composition of the ECM matters, as it drives differential eosinophil responses; (4) the provisional ECM is sufficient to promote a phenotype consistent with eosinophil tissue activation in allergic inflammation. Notably, the tissue composition of the lung in development is reminiscent of the lung in allergic eosinophilic asthma, which supports the notion that eosinophils may be driven by a common tissue morphogenetic program and ECM modifications in both development and disease.

Eosinophils are now emerging as inherently homeostatic innate immune cells associating with morphogenetic events and stem cell activity, as initially postulated in the LIAR hypothesis (eosinophils as regulators of ***L***ocal ***I***mmunity ***A***nd/or ***R***emodeling/Repair) by James Lee and colleagues^5^. In homeostasis, eosinophils can be found at sites experiencing high rates of epithelial shedding or turnover (small intestine, endometrial lining of the uterus, urinary bladder lining), during morphogenetic events (mammary gland duct branching morphogenesis), or stem cell activity (bone marrow, thymus)^5,12^. Similar to the mammary gland, the lung has also been shown to continue development after birth^13^. Our finding that eosinophils are driven by airway morphogenesis during the bulk septation and epithelialization phase of the alveolar stage of lung development supports the LIAR hypothesis and the emerging paradigm that Type 2 immunity evolved as a homeostatic response to assist with tissue remodeling and repair^25^. The use of new eosinophil-deficient PHIL (constitutive eosinophil knockout) and iPHIL (inducible eosinophil deficiency) mouse strains developed by Lee labs (Mayo Clinic Arizona) demonstrated that eosinophils are necessary for proper resolution and repair of the lung in a model of persistent allergic inflammation^26^. Moreover, eosinophils were found to promote larval growth by an IL-4-dependent mechanism and were necessary to protect host tissue against damage in a model of nematode infection^27^. Therefore, eosinophils may be engaged as part of Type 2 immunity to assist in tissue injury repair and morphogenetic processes. Eosinophils are not unique in this regard. Most innate immune cells linked to Type 2 responses (alternatively activated macrophages, innate lymphoid cells, mast cells) are now prescribed with novel functions associated with repair and remodeling. Group 2 innate lymphoid cells (ILC2s), mast cells, and basophils produce amphiregulin when they interact with the epithelium, which was shown to play a positive role in tissue repair^28,29^. Mast cells are also emerging as key players in the tissue remodeling and healing processes^30^. Moreover, cells found in lung tissue in chronic allergic disease express receptors for IL-33 and TSLP, which allows them to respond to epithelial barrier distress^31^. Epithelium and mesenchymal cells, in turn, express functional receptors for IL-4 and IL-13, effector Th2 cytokines, further supporting the existence of a two-way immune-tissue interaction. In this study, we examined one arm of this communication – tissue morphogenetic processes that may necessitate Type 2 responses in development and inflammation.

Neonatal immunity shows an inherent bias towards Type 2 polarization^17,32^. Cells of both the innate and adaptive immune system typical for Type 2-orchestrated inflammation have also been found to associate with normal lung organogenesis and development^9,12,33,34^. Monocytes and macrophages peak in the developing murine lung at the end of the saccular phase (embryonic day 18 to postnatal day 3)^17^. ILC2s, mast cells, eosinophils, and basophils are typically reported as peaking at various time points during postnatal days 3-14^2,15,17^, which would correspond to the primary (bulk) septation of the lung during alveolarization. Dendritic cells, T cells, and B cells significantly increase from postnatal day 14 and persist into adulthood^17^, which would correspond to the secondary septation of the lung and overall growth. M2-polarized macrophages localize to sites of branching morphogenesis and increase in number throughout the alveolarization stage of normal postnatal lung development^35^. Perinatal activation of the IL-33 pathway is frequently viewed as the main event promoting ILC2 accumulation in the neonatal lung, which in turn recruits eosinophils via the production of IL-5^15,17^. Alternatively activated macrophages have also been shown to be driven by IL-33 and to promote the lung tissue recruitment of eosinophils^15,36^. Despite these findings, the reasons for IL-33 activation and the presence of Th2 immunity in development are not known. First breath-induced lung tissue distress leading to the production of IL-33 as an alarmin^15^ followed by the possible involvement of ILC2s or alternatively activated macrophages^29^ is the only suggested upstream mechanism driving the accumulation of eosinophils in lung development. However, the protein levels of IL-33 are elevated immediately at birth and are sustained throughout the entirety of postnatal development^15^, while the eosinophil (as well as mast cell, basophil, and ILC2) kinetic clearly corresponds only to a time window between postnatal days 7 and 14. Even more intriguingly, when measured by gene expression, IL-33 actually increases at postnatal day 18 and thereafter is sustained into adulthood (data not shown). Overall, despite its relevance, an isolated focus on the IL-33/ILC2 axis represents a largely immune-centric (and downstream) view of shaping neonatal immunity, neglecting to address actual tissue morphogenesis as an upstream developmental event that collectively shapes the homeostatic immune response.

While mesenchymal activity and developmental pathways (WNT, Smoothened/Disheveled, Notch) are getting more attention in asthma and allergy research^37–41^, the functional contribution of the ECM to allergic disease pathogenesis and eosinophil biology is surprisingly overlooked. The ECM is not a passive structure but plays numerous critical regulatory roles in dictating the progression of morphogenetic events, recruitment and activation of hematopoietic and structural cells, and control of differentiation and fate of different cell types. Due to its functional importance, the composition of the ECM in tissue is highly regulated. Provisional matrix components are present at extremely low or negligible levels in homeostasis and only appear in the ECM during development or repair^42^. Our identification of a specific composition of the provisional ECM in mouse and human development and disease (enriched with tenascin-C, periostin, mindin, and thrombospondin 2) was exciting as it allowed us to more precisely characterize the biology of the tissue microenvironment associated with the Type 2 immune response and eosinophils. Interestingly, the signature that we describe here also matches the “Th2-high” subphenotype of asthma^43^. Top “Th2-high” genes with marker potential in allergic asthma are POSTN and SERPINB2. Both are ECM-associated proteins: periostin (POSTN) is a provisional matrix glycoprotein and plasminogen activator inhibitor-2 (SERPINB2) is an inhibitor of the plasminogen system that decreases matrix degradation and is implicated in tissue remodeling^44^.

Identification of tenascin-C both in mouse and human lung development and disease is even more exciting, as its expression is highly restricted in a spatio-temporally fashion, specifically localizing to the epithelial-mesenchymal interface during development or tissue regeneration^45^. Tenascin-C is a large, multi-domain glycoprotein that can simultaneously interface with multiple integrins on hematopoietic and structural cells, as well as other ECM proteins, such as fibronectin^46^. Eosinophil numbers were reported to correlate with tenascin deposition in atopic skin^47^ and eosinophilic esophagitis^48^. In mice, Tenascin-C deficiency impairs airway branching *ex vivo*^49^ and attenuates allergen-induced inflammation in a model of bronchial asthma^50^. The ECM protein mindin (spondin 2) identified in our study has also been independently shown to regulate the migration of eosinophils into the airspace in a model of allergic asthma^51,52^. Hyaluronan^53,54^, fibronectin^55,56^, and laminin^57^ have all been shown to regulate eosinophil recruitment and function, which warrants further investigation into matrix biology in allergic disease and its capacity to alter eosinophil accumulation and activity in human airways. Revisiting previous literature, it is apparent that not only eosinophils, but also basophils, mast cells, and ILC2’s exhibit a similar kinetic association with TNC deposition^17^. All of these cell types are known to associate with Type 2 immune responses and mucosal remodeling, which warrants further mechanistic investigation.

We also show that the exposure of naïve murine eosinophils to ECM substrates characteristic of development and remodeling is sufficient to upregulate markers typical for eosinophils sorted from tissue in allergic inflammation. Importantly, top markers of such tissue-activated effector eosinophils are integrins, among them CD11c, CD11b, CD103, and integrin beta 5. Given the specific upregulation of these integrins by tissue-recruited cells, eosinophils are well equipped to interact with various ECM proteins. Specific tissue ligands for these integrins are fibrinogen, fibronectin, and periostin, among others^1,58^. CD11c is a specific marker of eosinophil activation and is associated with a specific tissue subset of eosinophils targeted to the airspace during inflammation^1^. Interestingly, tissue resident eosinophils in the adult homeostatic murine lung are Siglec-F^med^CD11c^−^^1,2^. In allergic inflammation, this phenotype changes to Siglec-F^high^CD11c^+^ upon the trans-epithelial movement of eosinophils^1^. Remarkably, eosinophils in postnatal development exhibit a phenotype (Siglec-F^high^CD11c^+^) and morphology (segmented nuclei and presence of vacuoles (vEos)) characteristic of allergic inflammation, further suggesting that the similar reshaping of lung tissue in development and pathology commonly shapes eosinophil identity in both situations.

In view of the similarities between the tissue environments of development and disease, the lung tissue morphogenetic and extracellular matrix environment may represent “the iceberg” factor in setting the Type 2 immune response. Understanding the role of eosinophils in normal development will allow us to better understand the function or dysfunction of these cells in chronic tissue remodeling. Overall, placing eosinophils in the context of their immediate and dynamic tissue microenvironment is necessary to complete the picture of eosinophil participation in homeostasis and disease.

## Supplementary information

Supplementary Figures.

Supplementary Table 1.

Supplementary Table 2.

Supplementary Table 3.
