# Supplementary material for: Eosinophil accumulation in postnatal lung is specific to the primary septation phase of development
Source: Sci Rep. 2020 Mar 10;10:4425. doi: 10.1038/s41598-020-61420-5 (PMC7064572; doi:10.1038/s41598-020-61420-5)
Supplement: Supplementary file 1 — Supplementary Figures. [file 41598_2020_61420_MOESM1_ESM.pdf]

Eosinophil accumulation in postnatal lung is specific to the primary septation phase of development

SHORT TITLE: Eosinophils in lung postnatal development

AUTHORS: [1]Lucas F. Loffredo¥, [1]Mackenzie E. Coden¥, [1]Brian M. Jeong, [1]Matthew T. Walker, [2]Kishore Reddy Anekalla, [1]Ton C. Doan, [1]Raul Rodriguez, [1]Mandy Browning, [1]Kiwon Nam, [3]James J. Lee, [2]Hiam Abdala-Valencia and [1]Sergejs Berdnikovs*

[1]Division of Allergy and Immunology, Department of Medicine, Northwestern University Feinberg School of Medicine, Chicago, Illinois, USA

[2]Division of Pulmonary and Critical Care, Department of Medicine, Northwestern University Feinberg School of Medicine, Chicago, Illinois, USA

[3]Department of Biochemistry and Molecular Biology, Mayo Clinic Arizona, Scottsdale, USA

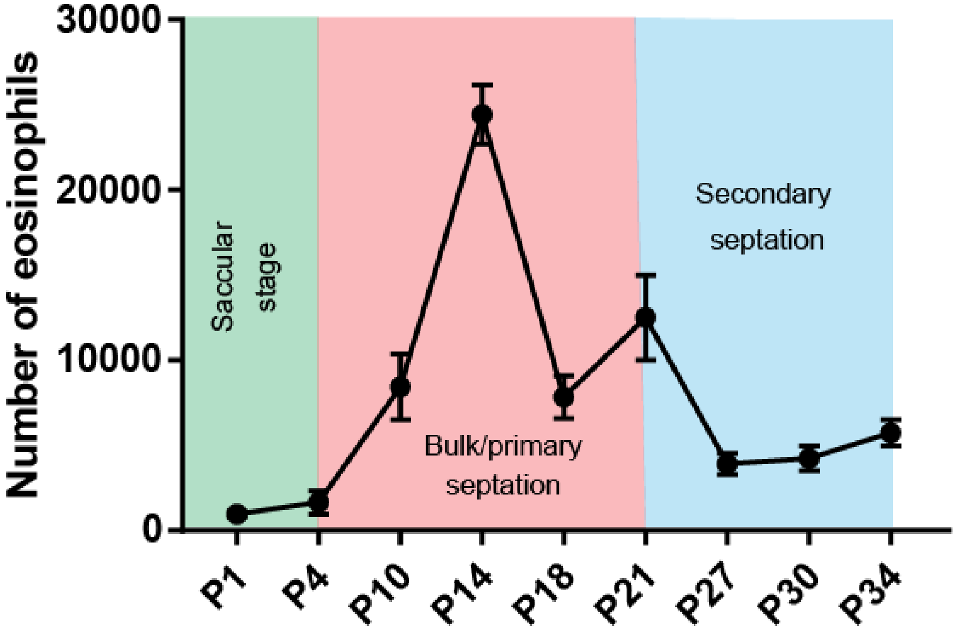

Figure S1. The total count of eosinophils in normal development matches the kinetic of eosinophil recruitment represented as percentage from CD45 cells shown in Figure 1A.

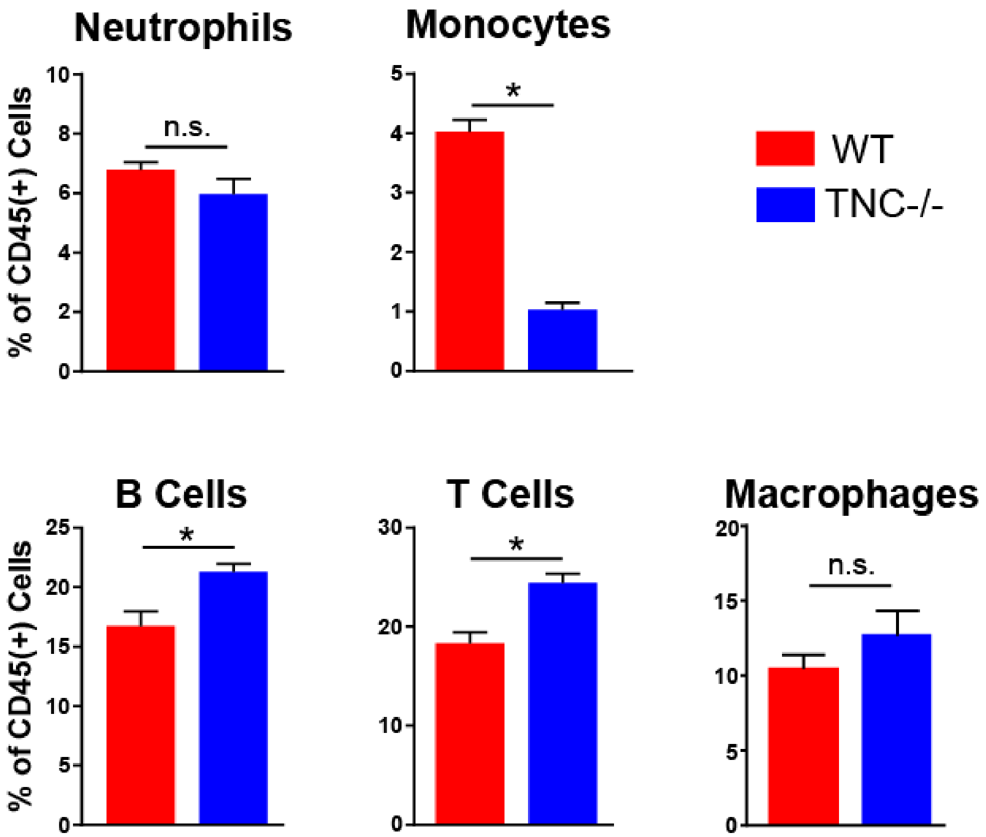

Figure S2. Leukocyte recruitment is altered in Tenascin C knockout mice as compared to wild-type mice.*, $p < 0.05$, unpaired T-test

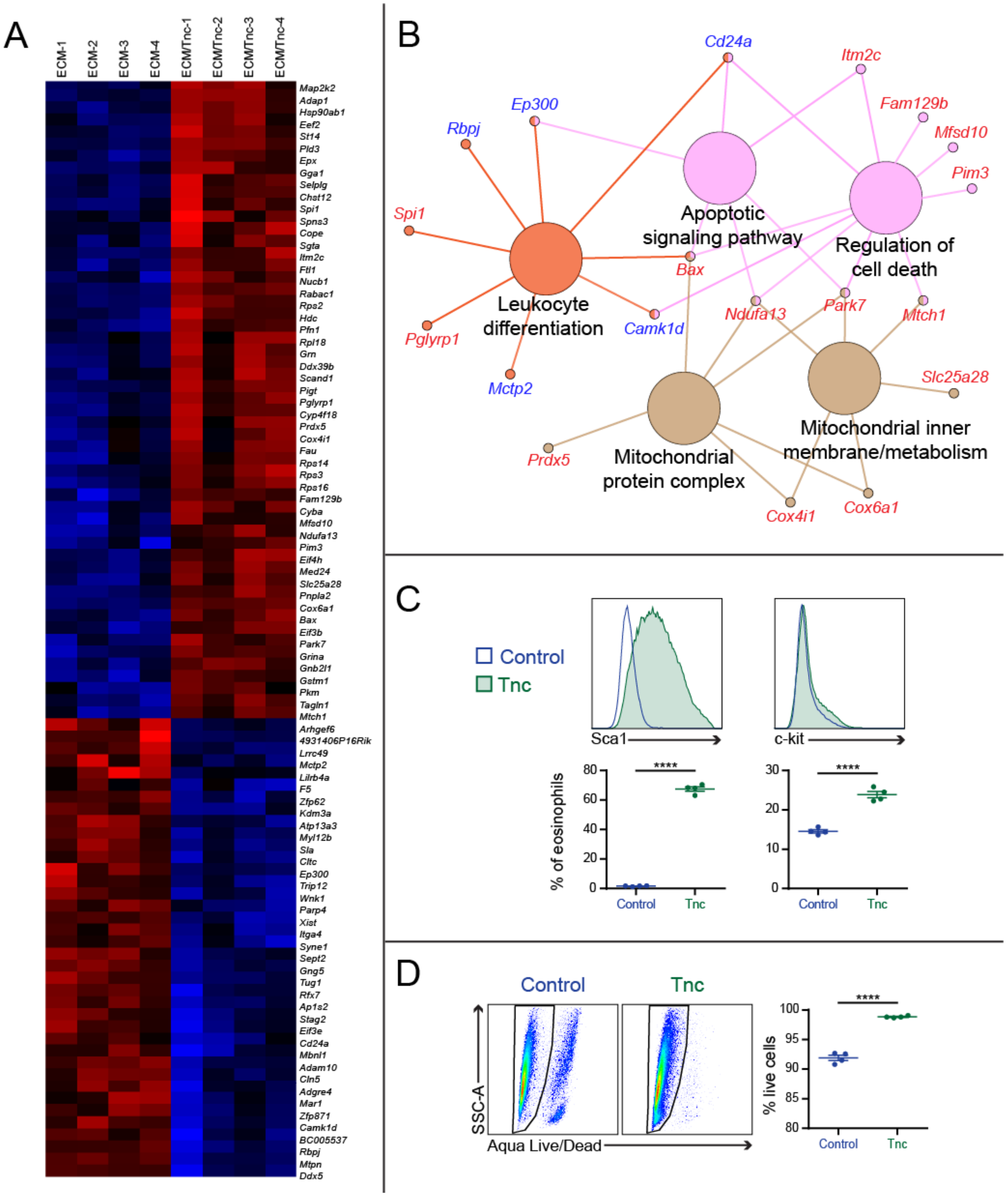

Figure S3. Gene expression profiles uniquely represented in eosinophils interacting with a tenascin-C-enriched extracellular matrix environment. A. Heatmap of 91 genes differentially expressed between ECM and ECM/Tnc treatments ($p_{adj} < 0.01$). *Red*: upregulated genes. *Blue*: downregulated genes. **B.** Functional network analysis of 91 gene signatures from A. **C.** Bone marrow-derived eosinophils

cultured from progenitor cells in the presence of Tnc (25 μg/ml) express significantly increased levels of Sca-1 and c-Kit, measured by flow cytometry as expression intensity and percent of eosinophils expressing these markers. ****, $p < 0.00001$, unpaired Student's t-test, N=4, representative of 3 independent experiments**.** **D**. Tnc-enriched eosinophils exhibit greater viability in bone marrow-derived cultures, measured by flow cytometry as a decrease in fluorescent live/dead dye incorporation. ****, $p < 0.00001$, unpaired Student's t-test, N=4, representative of 3 independent experiments.
